# Juvenile Dermatomyositis: New Clues to Diagnosis and Therapy

**DOI:** 10.1007/s40674-020-00168-5

**Published:** 2021-02-06

**Authors:** Lauren M. Pachman, Brian E. Nolan, Deidre DeRanieri, Amer M. Khojah

**Affiliations:** 1 Northwestern Feinberg School of Medicine, Divisions of Pediatric Rheumatology, Ann & Robert H. Lurie Children’s Hospital of Chicago, Chicago, IL, USA; 2 Cure JM Center of Excellence in Juvenile Myositis Research and Care, The Stanley Manne Research Center for Children, Chicago, IL, USA; 3 Division of Allergy/Immunology, Chicago, IL, USA, Ann & Robert H. Lurie Children’s Hospital of Chicago, Chicago, IL, USA

**Keywords:** Juvenile dermatomyositis, Myositis-specific antibodies, Myositis-associated antibodies, Infection, HLA specificity, Nailfold capillaroscopy, Therapy, Biomarkers

## Abstract

**Purpose of review:**

To identify clues to disease activity and discuss therapy options.

**Recent findings:**

The diagnostic evaluation includes documenting symmetrical proximal muscle damage by exam and MRI, as well as elevated muscle enzymes—aldolase, creatine phosphokinase, LDH, and SGOT—which often normalize with a longer duration of untreated disease. Ultrasound identifies persistent, occult muscle inflammation. The myositis-specific antibodies (MSA) and myositis-associated antibodies (MAA) are associated with specific disease course variations. Anti-NXP-2 is found in younger children and is associated with calcinosis; anti-TIF-1γ+ juvenile dermatomyositis has a longer disease course. The diagnostic rash—involving the eyelids, hands, knees, face, and upper chest—is the most persistent symptom and is associated with microvascular compromise, reflected by loss of nailfold (periungual) end row capillaries. This loss is associated with decreased bioavailability of oral prednisone; the bioavailability of other orally administered medications should also be considered. At diagnosis, at least 3 days of intravenous methyl prednisolone may help control the HLA-restricted and type 1/2 interferon–driven inflammatory process. The requirement for avoidance of ultraviolet light exposure mandates vitamin D supplementation.

**Summary:**

This often chronic illness targets the cardiovascular system; mortality has decreased from 30 to 1–2% with corticosteroids. New serological biomarkers indicate occult inflammation: ↑CXCL-10 predicts a longer disease course. Some biologic therapies appear promising.

## Introduction

Juvenile dermatomyositis (JDM) is a rare disease with a characteristic rash and symmetrical proximal muscle weakness. Although it is classified as a vasculopathy [1], pharmacokinetic data suggests that the microvascular damage may not be limited to the skin [2] and muscle [3] and also may include the vasculature of the gastrointestinal tract, with a considerable negative impact on the bioavailability of some orally administered drugs [4]. The goal of this communication is to acquaint the reader with the common symptoms of this rare disease and provide current information concerning diagnosis and therapy [5]. Increasing clinical national and international collaboration [6] has facilitated the generation and analysis of larger datasets. There are more specific guidelines for diagnosis [7] and criteria for improvement [8], which are useful both for the western world and for the practitioners in China [9] and Japan [10]. This specific documentation has provided new clues concerning JDM pathophysiology, widening the spectrum of potential medical therapies.

## Juvenile dermatomyositis: symptoms and signs at diagnosis

The annual incidence of JDM by race in the USA is 3.4/million for white, non-Hispanic, 3.3/million for African Americans, and 2.7/million for Hispanic patients, with an overall girl-to-boy ratio of 2.3 girls:1 boy [11]. For the USA as a whole, the mean age at JDM diagnosis is 6.7 years for girls and 7.3 years for boys [12]. In our clinical population, which includes more than 500 children with definite/probable JDM [13, 14], 35.2% are under the age of 5 years at entry. The cutaneous features include Gottron’s sign—linear erythema–localized to the areas of the hands where the skin is stretched, over joints, while Gottron’s papules usually occur in areas of injury—fingers, elbows, and knees. Not only does the shawl sign occur on the upper chest, but also a typical rash may occur on areas of the body where there is pressure on the skin, such as the belt line. In addition to the classic rash and symmetrical proximal muscle weakness, initially identified by Bohan and Peter [13, 14], untreated children are shorter and lighter than their peers [15] and may develop the following complications: interstitial lung disease [16]; edema, either localized or general—secondary to capillary leak; and hair loss—secondary to scalp inflammation/edema. Cardiovascular damage includes impaired cardiac conduction [17, 18], and subsequent cardiac systolic dysfunction is predicted by cutaneous inflammation [19]. Evidence of microvascular damage can be documented by loss of capillary end row loops (ERL) in a child’s nailfold/periungual area [20, 21]. A well recognized physical finding—the presence of dilated capillaries at the eyelid margin—is often the last cutaneous sign to disappear [22]. Bone density is decreassed in the untreated child with JDM and associated with an increased RANKL:osteoprotegerin ratio [23]; lipodystrophy is also a consequence of the JDM inflammatory process [24]. The onset of the characteristic rash, and the defining features of muscle damage with symmetrical proximal weakness, can run the gamut from insidious to very rapid progression.

The duration of untreated disease (DUD), the interval between the first symptom and the start of therapy, is a critical factor in understanding disease pathophysiology, impacting both the clinical findings and diagnostic laboratory data. Weaker children come to diagnosis earlier in their disease; those with only a mild rash—often much later [15]. The standard muscle enzymes are severely impacted as well (see serologic testing). As expected, gene expression profiles of muscle tissue are also modified by the DUD [25], which is noted infrequently in patient profiles.

### Circumstances surrounding the onset of JDM

A variety of physical conditions may contribute to the development of JDM, including exposure to ultraviolet light [26]; the genetics of the patient appears to be associated with susceptibility to the increased dosage of UVB at equator-related latitudes [27]. Higher mean UV index exposure was associated with increased odds of using biologics or non-methotrexate disease-modifying anti-rheumatic drugs and skin ulceration, as well as developing calcinosis [28]. Increasing particulate pollution in the city atmosphere inflicts damage to the developing fetus and later in life [29]. Air pollution provokes oxidative stress, fostering the increasing development of a spectrum of autoimmune diseases [30], including JDM [29]. Exposure to an infectious agent may also play a role in precipitating the symptoms of JDM. A national study of 323 newly diagnosed children with JDM documented the presence of an antecedent illness, often in the upper respiratory tract, in the 3 months before JDM symptoms were noted [12].

Studies of *seasonality of birth* of children who later developed JDM [31] were associated with spring and fall seasonality of JDM onset in the Midwestern United States in the early 1980s. At the same time, there was an increase in both complement fixing and neutralizing antibody to Coxsackievirus B2 and B4 in newly diagnosed, untreated children with JDM [32]. Parvovirus 19 has also been implicated [33] as well as other agents. This apparent seasonality influence was lost, coincident with the increasing impact of global warming in about the mid-1980s, which stimulated a comprehensive re-evaluation of the impact of the accelerated rate of climate change [34]. An independent analysis of our gene expression profile data from muscles from children with untreated JDM recently concluded that the response to a double-stranded RNA agent [35] might be sufficient to initiate the interferon-based inflammatory progression typical of JDM [36].

The exact factors underlying the induction of the autoimmune response in JDM are not known, although multiple lines of evidence support the concept that *an environmental exposure triggers disease in genetically susceptible individuals*. Disease susceptibility has been mapped to the HLA locus on chromosome 6 using the genome-wide association methodology in a European population [37], [38]; C4A null alleles [39] and HLA-DRB1 03:01 [38] are both risk factors for JDM. Differences in HLA associations with anti-TIF-1γ (p155/140) autoantibodies have been identified in adult-onset vs juvenile-onset DM [40], which may account for the differences in the disease course in these two otherwise similar populations. Increased CD4+ cells, TH-17, IL-6 [41], an expanded B cell population [42], and the emerging role of natural killer cells [43], as well as the PTPN22 R620W variant, are all linked to the increased risk of developing juvenile dermatomyositis.

### Diagnostic testing overview

A wide spectrum of specific assessments has been compiled to aid the physician to evaluate skin and muscle involvement in both children and adults with inflammatory myopathies: JDM, juvenile polymyositis, DM, PM, and inclusion body myositis (IBM) [44]. A patient-reported outcomes system (PROMIS) has been developed for the age range of JDM disease onset—5–7 years of age—and found that a parent report cannot be substituted for the disclosure by the child [45]. A preliminary approach to evaluation of children with JDM was discussed using this PROMIS methodology [46].

## Serologic and other blood assays

- Muscle enzymes: the standard diagnostic muscle enzymes (LDH, SGPT) normalize by 2.53 and 3.68 months respectively after symptom onset, while CPK and aldolase normalize by about 4.5 months [15]. Therefore, although CPK is a mainstay of the international classification criteria [7], it is not reliable after about 4 months between the first symptom and the clinical evaluation.
- Other “routine initial” testing: ANA (usually speckled), myositis-specific antibodies and myositis-associated antibodies, CBC, differential, CRP (elevated in overlap syndrome but not usually in JDM), ESR (usually normal range), U/A, BUN, Cr, neopterin—for macrophage activation; flow cytometry (for levels of both B cells and natural killer cells), C4 (for C4 null alleles), von Willebrand factor antigen (and blood group for normal ranges), vitamin D, DXA, and nailfold (periungual) capillary end row loop data [21].
- Myositis-specific antibodies (MSA) [47-50] (Table 1) vs myositis-associated antibodies (MAA) (Table 2) [48]: The importance of the expanding list of these antibodies cannot be overstated, for they provide a testable rationale for the heterogeneity of the inflammatory myopathies [51] in both adults and children [52, 53]. The most common type of MSA is antibody-TIF1-γ (transcriptional factor-1-γ) which is also called p155/140, occurring in 18–30% *of children* with JDM, and associated with a more prolonged and severe disease course [54], [55]. In over 25% *of adults* with inflammatory myopathy, the detection of both TIF1-γ and anti-NXP-2 (also called anti-MJ) is associated with the occurrence of a malignancy within a year of inflammatory muscle disease diagnosis [56]. Anti-NXP-2 has, as its target, NXP-2 and occurs in 15–22% of the children with JDM; it is associated with calcinosis, especially in children diagnosed below the age of 5 years [57] who manifest more severe muscle disease and gastrointestinal bleeding, resulting in a worse disease outcome with a lower functional status [58].

Overall, the next most common MSA is melanoma differentiation-associated gene 5 (MDA-5). In Japanese children, the frequency of MDA-5 is increased to 28% [59] vs 6% in the western world [52]. In China, the frequency of MDA-5 is similar both in children and in adults—30%—and associated with an amyopathic disease course in DM, interstitial lung disease (ILD), and digital ulcers [60]. A new biomarker, Krebs von den Lungen-6 (KL-6), appears to be useful in identifying children with ILD and is associated with increased IL-18 and ferritin [61]. ILD is increased in DM patients positive for MDA-5 who also may be clinically amyopathic (CADM) [51], as well as in children with inflammatory myopathy and other MSA, but can be reversed by aggressive therapy [62]. IV corticosteroids and cyclosporine A are frequently used for JDM with ILD; some biologics are promising.

The clinical picture is further complicated by the concurrent presence of myositis-associated antibodies (MAA), identifying overlap syndromes, such as those associated with antibodies to ribonuclear proteins (Table 2). Children positive for MAA follow a more chronic and relapsing disease course and have an increased frequency of calcinosis. An MAA, anti-Ro-52, is associated with ILD in children with JDM [63]; children positive for ILD also have increased levels of neopterin [16] which should prompt the use of high-resolution computerized tomography (CT) or ultrasound—to scan the lungs—as well as pulmonary function testing, if over the age of 6.

## Diagnostic testing

### Muscle strength and endurance

CMAS and MMT-8 are the mainstay of most clinical evaluations of muscle strength and endurance in children with JDM. To document the physical impairment consequent to the symmetrical proximal and truncal muscle weakness, it is very helpful to have an independent assessment by a physical therapist in a clinic at the time of a physician’s physical exam. However, the standardization of CMAS, to achieve a score of 52, was designed for healthy children aged 4–9 [64]. In contrast, for healthy children aged 4–5, we found that the achieved score was much lower, at 46, not 52 [65]. Over 26.3% of our newly diagnosed JDM are under age 4 at their first visit; interpreting CMAS data is hampered by lack of normal standards for younger ages.

## Computerized tomography

### Interstitial lung disease

This modality is used extensively in evaluating the presence and progression of ILD in JDM. Anti-Ro-52 antibody is associated with ILD in children with JDM [63] (see MSA/MAA above).

### Volume of calcifications

A challenge in the treatment of JDM calcifications is to obtain convincing evidence that a medication is “working”—that the calcification volume is decreasing. Calcifications may respond slowly to immunosuppression or not at all. Single-slice CT as well as ultrasound (see below) can document the change in the volume of the calcifications as it grows or, in response to therapy, diminishes in size [66].

### MRI: verification of target tissue involvement

Not only is MRI the method of choice for documenting inflammation and damage in all the proximal muscles at once, but it is also useful in evaluating unsuspected subcutaneous fat and fascial involvement [67]. When whole-body MRI (WB-MRI) utilizing short tau inversion recovery (STIR) was used to evaluate symptomatic and asymptomatic areas in muscle, subcutaneous tissue, and myofascial areas in 41 JDM patients, unsuspected areas of involvement were documented, and the pattern on MRI varied from patchy to diffuse [68]. This assessment is useful to identify persistent areas of inflammation, but in the young child with JDM, sedation is often needed, and the expense of the method is a deterrent.

### Ultrasound in JDM

There is an emerging role for ultrasound in the evaluation of myositis in children with JDM. Clinical and laboratory parameters do not always parallel disease activity, so other methods of evaluation, such as muscle ultrasound, can provide complementary data in the evaluation of myositis [69]. Muscle thickness and echogenicity, which describes the ability of a tissue to reflect a sound wave, have been proposed as means of assessing disease activity in JDM [70]. In acute disease, muscle inflammation and edema are associated with an increase in muscle size and decrease in echogenicity, respectively, whereas in chronic disease, fatty infiltration and muscle atrophy are associated with decreased muscle size and increased echogenicity [70]. A significant correlation was found between echogenicity, CMAS, and serum CK [70]. Both fasciitis and calcinosis are also easily visualized, measured, and monitored by ultrasound [71], [72]. MRI is better suited to the initial JDM investigation given its ability to evaluate all the proximal muscles at once. Monitoring disease activity, on the other hand, may be more easily achieved with ultrasound. It is more readily available, transportable, cheaper, and non-invasive compared to MRI and can be performed as a point-of-care service in clinic. Changes in muscle thickness and muscle echogenicity are readily assessable by ultrasound. Ultrasound has a superior resolution and can detect more subtle changes in the muscles than MRI. In one study, which included seven children with JDM, ultrasound detected changes in the muscle fiber 1 year after whole-body MRI had normalized [69]. Thus, ultrasound may be a more sensitive measure of change and positive changes in the context of a negative MRI may be significant. For example, the presence of subclinical myositis may explain why some patients have weakness despite having a normal MRI and muscle enzymes [69].

Newer applications of ultrasound have explored vascularity within the muscles as a measure of disease activity and include the use of power Doppler (PDS), contrast-enhanced ultrasound (CEUS), and sonoelastography [73], [74]. Both power Doppler and color Doppler are used to evaluate blood flow and hyperemia within tissue. Color Doppler can also detect directional flow and can thus differentiate between arterial and venous circulation. In CEUS, microbubbles are infused intravenously and travel to areas of high perfusion, such as inflamed skeletal muscles. The degree of perfusion can be measured by CEUS and thereby serve as another marker of disease activity [75]. Sonoelastography takes advantage of the mechanical properties of tissues to assess inflammation, with stiffer tissue being more suggestive of inflammatory myositis [73]. Using these imaging modalities, the optimal site for biopsy can also be targeted, and ultrasound-guided muscle biopsy can be used to obtain the tissue. Current advances in adult myositis include developing machine learning methods to classify myositis by detecting changes in echogenicity of the affected tissue (muscle, fascia, subcutaneous tissue, fat, etc.) and correlating these results with different disease subtypes, for example, DM, PM, or IBM [76]. While computational approaches are emerging for classification purposes [77], machine learning in the context of ultrasonography has not been applied JDM, yet.

### Increased multiple autoimmunity in JDM

Polyautoimmunity is not-uncommon in a person with a well-defined autoimmune disease [78]. The treating physician should consider screening for additional diagnoses in JDM patients with incipient symptoms, especially those treated with immunosuppressive medication. Immunosuppression may partially mask another emerging autoimmune disease, which occurs at higher than average rates in the families of patients with juvenile idiopathic arthritis, childhood lupus, and juvenile dermatomyositis [79]. In our study which evaluated familial autoimmunity within a three-generation pedigree of patients with JDM, SLE was reported in 50.1% of families [80]. A prospective analysis of our cohort of 590 JDM registry patients at the Ann & Robert H. Lurie Children’s Hospital of Chicago documented that the frequency of multiple autoimmunity is 8.7% (excluding diagnoses such as lupus or scleroderma that would be more representative of an underlying diagnosis of overlap syndrome). The additional autoimmune problem is most commonly vitiligo or psoriasis (*n* = 9 each), celiac disease (*n* = 8), and Hashimoto thyroiditis (*n* = 4). Four such patients have 2 additional autoimmune diagnoses, and one child with JDM has 5 additional diagnoses. Multiple autoimmunity has been described in smaller cohorts of JDM patients, with Hashimoto thyroiditis reported most frequently [78]. Multiple autoimmune diseases are a common feature of patients with diverse underlying syndromes of immune dysregulation including APECED (due to mutations in AIRE), CTLA-4 or LRBA haploinsufficiency, and Stat3 gain of function.

### Accessible serum biomarkers

Many physicians are familiar with the phrase “clinically quiescent but immunologically active” which makes tapering of medications difficult. There is an urgent need for accessible serum biomarkers which can alert the physician to ongoing disease activity—despite the fact that the JDM appears to be “inactive”—both to guide therapy and to explore more effective interventions. A series of recent studies have emerged, ranging from a review of biomarkers of calcinosis [81] to studies of possible novel mechanisms propelling their formation [82]. A comprehensive series of investigations have identified serological indicators reflecting endothelial and vascular disruption [83-85]. For example, endoglin elevation and a low level of sICAM-1 were associated with loss of the end row loops (ERL) in the nailfold (periungual) area of untreated children with active JDM [85]. Loss of ERL is clearly associated with *decreased bioavailability of orally* administered prednisone compared to the same dose, given as an IV preparation [4]. Elevated levels of galectin 9, galectin 1, TNFR-2, and CXCL-10 indicted the need for *intensification of treatment* within the first 3 months, while high CXCL-10 at diagnosis also appeared to be predictive of a longer time to drug-free remission [85]. A recent study, using the Soma™Scan technology, identified serum proteins in JDM at various stages of therapy (untreated, on maximal therapy, and when being tapered) which are as follows: (a) responded to therapy, (b) rebounded when therapy was decreased, or (c) never responded to therapy at all—opening up new areas of investigation [86].

## Treatment options

Although there is a fair amount of diversity in a physician’s selection of therapy [87], disease control can usually be obtained with currently available medications, which we hope has improved with the CARRA guidelines for care (see below). Of note, allogeneic bone marrow transplantation, sometimes used to treat inflammatory myopathy, can also elicit symptoms of inflammatory myopathy [88].

### Physical therapy

The ventilatory capacity is often impaired in children with JDM because their truncal muscles are weakened. This muscle weakness contributes to low bone mass, aerobic deconditioning, and exercise intolerance. In contrast to the prolonged bed rest initially recommended for new-onset JDM, a randomized control trial of 26 JDM that variably assigned a 12-week home-based exercise program concluded that children who exercised had greater improvements than controls; but isometric strength and perception of fatigue were unaffected; no one required an increase in medications or hospitalization [89]. Exercise also helps reduce depression and assists in the recovery of muscle strength [90].

### Medical therapy

Prior to corticosteroids, one third of the children diagnosed with inflammatory myopathy (JDM included) had calcinosis, one third died, and one third survived [91]. The scarcity of JDM patients led to the spontaneous use of a range of drugs. In contrast, a recent succession of useful protocols—which gauge the child’s response to therapy—has emerged from the Childhood Arthritis & Rheumatology Research Alliance (CARRA) group of pediatric rheumatologists. The initial guidelines for the treatment of moderately severe JDM [92] were followed by specific clinical treatment plans for JDM beyond the first 2 months [93], a protocol for the treatment of skin-predominant disease [94] as well as guidelines for the treatment of JDM with a persistent rash [95]. Of note, the actual usage of the medications to treat 320 children with JDM was recently reviewed [96].

### Vitamin D

Inasmuch as dietary sources of vitamin D provide only 10% of the needed vitamin D and the recommended blood level for vitamin D for children is 30 IU or above, a daily supplementation with vitamin D of 1–2000 IU is recommended [97]. Low serum levels of vitamin D occur in people who have the idiopathic inflammatory myopathies (IIM) [98] including children with JDM [99]. Low levels are also found in subjects who are of increased age, are female, have a darker skin tone, and live in the northern latitude and stay there through the winter season of the year [97]. Since JDM often flare with sun/UVB exposure, they are usually sequestered during times of maximum exposure (see “Other therapies” below) and supplementation with exogenous vitamin D is required. Vitamin D deficiency is inversely associated with parathyroid hormone levels, which, as they rise, increases the risk of cardiovascular damage [100].

### Corticosteroids

The mainstay of JDM medical therapy remains to be corticosteroids [101] which are life-saving, decreasing mortality to from 30 to 5% or much less [101, 102]. It is now customary to start with high-dose intravenous methyl prednisolone (maximal at 30 mg/kg), given in three or more consecutive daily doses once the diagnosis of JM is confirmed, as documented in a multinational PRINTO study [103]. Adverse reactions to IVMP in children are variable and encompass hyperglycemia, GI distress, hirsutism, breast enlargement in males, moon facies, thin skin with purple striae, osteoporosis, and CNS irritability, which includes an uncontrolled rage reaction [104]. Of note, absorption of oral prednisone is impaired in JDM patients whose nailfold end row capillary loops (ERL) fall below the normal range of 7 down to 5 ERL or less/3 mm [4]. This limitation was not considered in the recent recommendations by PRINTO for tapering of corticosteroids over time [105] and may vary with different medication preparations/conditions. Maintenance doses of corticosteroids range from 2 mg/kg/day to incrementally less. Gastric protection when corticosteroids are given is essential; options range from H2 blockers and proton pump inhibitors, to barrier protection such as Gaviscon^®^. The consequences of steroid therapy also include increased susceptibility to *Pneumocystis jirovecii* [106].

### Hydroxychloroquine

A screening eye exam is required before starting this drug. The Academy of Ophthalmology Guidelines for testing for hydroxychloroquine retinopathy were changed from an “annual” exam to “5 years after starting the drug” [107]. The recommended dosage is 5 mg/kg/day; adverse reactions include abdominal pain ± nausea, which is the most common complaint, as well as cardiomyopathy, cardiac arrhythmia, elevated liver function tests, bone marrow suppression, and myopathy. Hydroxychloroquine is often not effective as a single drug [108]; it did not appear to improve the rash of 184 children with JDM [22]. The effect of hydroxychloroquine on the immune system includes increasing the lysosomal pH in antigen-presenting cells, resulting in decreased production of IL-1, IL-6, and TNF-α as well as blocking TLR7/9 on plasmacytoid dendritic cells, thus reducing the type 1 interferon signature which drives the inflammatory response [109]. In addition to its considerable anti-thrombotic effect [110], hydroxychloroquine is associated with a significant reduction in total cholesterol, triglycerides, and LDL levels [109] and is widely used for this purpose [111].

### Methotrexate

The introduction of methotrexate after 1997 has allowed the use of less prednisone for disease control [96], [112]. The dosage is usually 1 mg/kg or 15 mg/m^2^ with a maximum of 25 mg/week, preferably given subcutaneously, accompanied by 1 mg/day of folic acid (on days other than the day that methotrexate is given) [113]. The most common adverse reaction is nausea and vomiting. Hair loss, which can be severe, and mouth sores, followed by bone marrow suppression, lung disease, lymphomas, and acute renal failure, are noted, but much less common [114]. Pregnancy is a contraindication; drug contact is associated with fetal malformations and miscarriage. High-risk patients should be screened for hepatitis B and C serology and if present, another drug should be used [114]. A recently identified mechanism of action is pro-respiratory and anti-growth by promoting AMPK signaling [115]. Withdrawal of this drug should be considered if the child is off steroids and in remission for at least a year.

### Mycophenolate mofetil

Mycophenolate mofetil (MMF) and Myfortic should not be used interchangeably due to differences in concentration and absorption. Mycophenolate mofetil is given every 12 h at 20 mg/kg (maximal doses of 1000 mg/24 h). MMF is associated with congenital malformations and should be discontinued 7 weeks before a planned pregnancy. For girls, urine for pregnancy testing should be obtained at every visit when appropriate. This drug is a reversible, selective, and non-competitive inhibitor of inosine monophosphate, a critical enzyme in the de novo purine synthesis pathway required for lymphocyte proliferation [116]. At the moment, there are no guidelines to help identify who will benefit from MMF [117]. A study of 50 JDM patients documented that MMF both was steroid-sparing and decreased muscle and skin inflammation without a drop in the white count or an increased number of infections [118]. Adult DM with interstitial lung disease responded well to MMF. Although it is not widely recognized, vitamin D can lower the effective dose levels of MMF [119] which is a cause for concern: maintaining levels of vitamin D in a therapeutic range is critical for the well-being of children with JM, but unfortunately, a reliable testing for MMF levels is not widely available. Other adverse effects of MMF include diarrhea, bone marrow suppression, and reactivation of hepatitis B and C—children at high risk for hepatitis should be screened before the drug is given.

### Tacrolimus

Much less used in recent years [120], [121], this agent has been effective, when combined with corticosteroids, in the treatment of a very difficult, relatively new form of inflammatory myopathy, defined by antibody to 3-hydroxy-3-methylglutaryl-coenzymeA reductase, and designated as HMGCR [122].

### Intravenous immunoglobulin therapy

The half-life of intravenous immunoglobulin therapy (IVIG) is 3 weeks; the drug is usually given at 1–2 g/kg every month. One mode of action appears to be the induction of autophagy in peripheral blood mononuclear cells, thus reducing the production of inflammatory cytokines [123]. Hyaluronidase-facilitated immunoglobulin can be given subcutaneously at much higher doses [124, 125]. Tests for both immunoglobulin A (IgA) deficiency and myositis antibodies (MSA/MAA) should be obtained prior to the administration of the monomeric immunoglobulin, which is usually given intravenously. Reactions to IVIG, such as flushing, flu-like symptoms, headache, and fatigue, commonly occur about 24 h after the infusion and may last as long as 3 days, interfering with school attendance. Established guidelines suggest that IVIG should only be given to patients who have treatment failures or those who have become corticosteroid-dependent. The use of IVIG for interstitial lung disease was not recommended. There are a range of mechanisms proposed for the action of IVIG: cytokine and autoantibody neutralization, saturation of the Fc receptor, blocking receptor activation—which includes binding to anti-inflammatory receptors as well as other modes of cell modulation [124, 125]. Its use is advocated for JDM children who initially had both muscle and skin involvement but who continue to have rash as a persistent, but not predominant, complaint [95].

### Cyclosporine A

This potent drug is a second-line agent and is given only when other, less toxic drugs have failed—in corticosteroid-resistant JM, often JDM with persistent rash [126]. A recent randomized trial of 139 JDM patients compared 3 treatment plans in 22 countries: after an induction period consisting of three daily doses of IV methylprednisolone at 30 mg/kg (1 g = maximal dose), the children were given either 2 years of oral prednisone alone vs prednisone + methotrexate vs prednisone + cyclosporine A (CyA). The study concluded that combined prednisone + either methotrexate or CyA performed better than prednisone alone, but more adverse reactions occurred with CyA (given at 4–5 mg/kg) requiring discontinuation of the drug [96]. In children with JDM, CyA can be used successfully at lower doses, 3 mg/kg, to maintain an 11th hour trough level of 80–110 ng/ml. An inhibitor of T cell activation, the pro-drug, CyA becomes activated after complexing with cyclophilin. This intracytoplasmic protein complex then inhibits calcineurin, a phosphatase that mediates the pharmacologic effects. Lipophilic in nature, a raised serum lipid level increases clearance of the drug, which therefore should be given before mealtime. Correspondingly, testing CyA levels should also performed before the child has eaten [55].

### IV cyclophosphamide

This drug has been used successfully to treat refractory JDM, at a dosage of 500/m^2^ q 2 weeks ×3, then 750 mg/m^2^ q 3–4 weeks for 3–4 session [127]. Unfortunately, cyclophosphamide (CYC) induces oxidative stress and has a highly destructive effect on the reproductive organs. For ovaries, this can be averted by modern methods of ovary removal and storage with later reimplantation when therapy has been attenuated [128]. The cardiotoxic effects can also be muted by antioxidant supplementation [129].

### Biologic agents: TNF inhibitors, rituximab, abatacept, and tocilizumab

Biologic agents are emerging as the targeted therapy for children with juvenile dermatomyositis [130] as well as other idiopathic inflammatory myopathies [131]. The possible immune pathways and their potentially specific drug targets in myositis have been recently reviewed [132].

### TNF inhibitors

While these drugs are very useful in the treatment of other rheumatic diseases [133], this class of drugs is far from optimal in the treatment of inflammatory myopathy [134]. Another problem is that the use of TNF inhibitors to treat other rheumatic diseases, such as psoriatic arthritis, has precipitated the development of JDM [135], as well as inflammatory myopathies in adults [136, 137].

### Rituximab

**Fig. 1.**
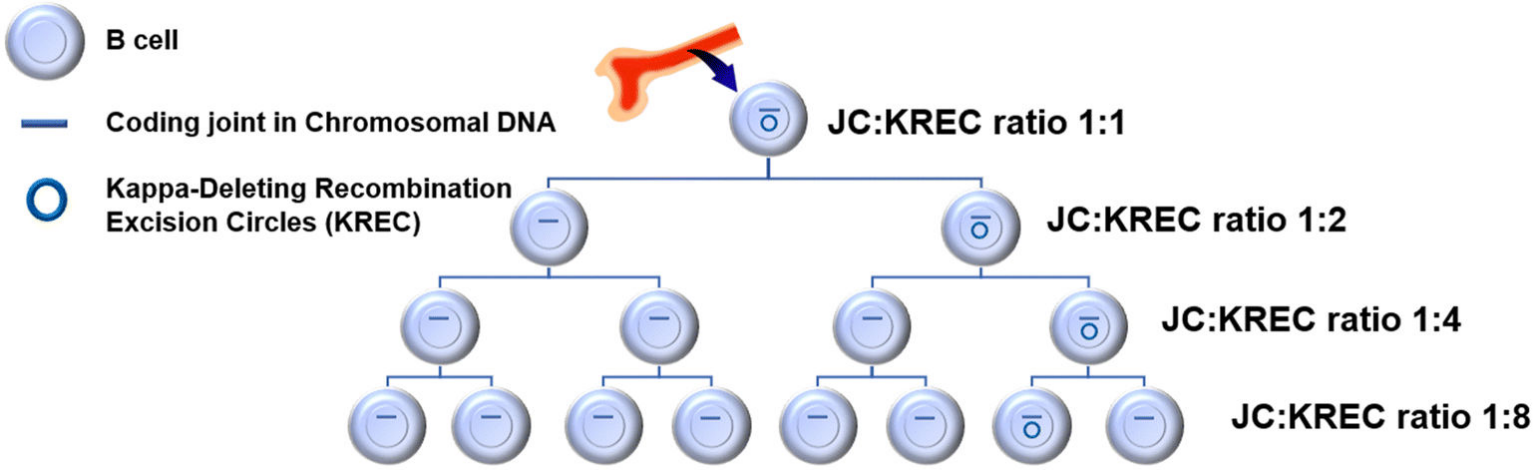
Cartoon of the kappa-deleting recombination excision circles (KREC) decreasing with cell division. The B cells originate in the bone marrow, located in this figure above the B cells. Khojah AM, Marin W, Morgan GA, Pachman LM. Kappa-deleting recombination excision circles (KREC) in B cells and serum B cell activating factor (BAFF): possible aids in predicting juvenile dermatomyositis response to rituximab. *Arthritis Rheum* [Internet]. 2018; 70(Suppl 10) abstract #11

A monoclonal antibody directed against CD20, a surface marker for B cells, rituximab results in B cell depletion via several mechanisms which include complement fixation, antibody-dependent cellular cytotoxicity, and signaling of apoptosis. A close monitoring of the serum immunoglobulin G (IgG) levels is advisable, especially in the first year of therapy, for there is a 30–50% prevalence of hypogammaglobulinemia; some patients require an immunoglobulin replacement therapy [138]. Rituximab has been used successfully to treat JDM patients who failed first-line therapy [139, 140], [141]. A patient’s response to rituximab can be assessed by identifying the efficiency of bone marrow replacement of B cells. Forming new B cells requires B cell receptor recombination, which leads to the formation of KREC, a unique small piece of circular DNA that does not replicate as the B cells divide, so the KREC remains only in one cell and not the progeny (see Fig. 1). The site of recombination on the chromosomal DNA after the excision of KREC is known as the “joining code” (JC), which is found in all B cell progeny. The ratio of JC to KREC determined by qRT-PCR estimates with precision the number of B cell divisions that have occurred in the child’s B cell population. Therefore, it can provide a clue for the degree of peripheral B cell depletion [142]. The initial reports were followed by large multicenter randomized controlled clinical trial to evaluate the effectiveness of rituximab in both adult and pediatric patients with refractory myositis [139, 140], including cutaneous improvement [141]. Although the trial failed to show a significant difference between the study groups, post-study analysis suggested that specific MSA (anti-Jo-1 or anti-Mi-2) or young age (juvenile dermatomyositis vs adult myositis) predicted a better response to rituximab and that the most common side effects were infusion reactions, infections, and low blood counts [141]. We have found that those JDM patients who are treated with rituximab with complete or near complete B cell depletion will have a low JC-to-KREC ratio (see Fig. 1) which is associated with a more effective response to rituximab [143].

### Abatacept

This genetically engineered fusion protein is constructed from the FC portion of IgG1 and the extra-cellular domain of the cytotoxic T cell lymphocyte-associated protein 4 (CTLA-4). This drug decreases T cell activation by blocking the 2nd signal—CD80 and CD85—from antigen-presenting cells such as B cells [142]. Reports of adults with IIM who are not doing well on other treatments responded to abatacept and documented that the use of this drug along with methotrexate improved their outcome [118]. Used in conjunction with thiosulfate, abatacept was successful in eliciting regression of calcific lesions [144]. Of note, Dr. Rider at the NIH is still recruiting patients to complete their drug trial on abatacept in children with JDM and resistant calcinosis.

### JAK kinase inhibitors

A dominant inflammatory pathway in JDM utilizes the type 1 interferons; their production is blocked by JAK kinase inhibitors. Tofacitinib blocks JAK 1 and 3, while baricitinib and ruxolitinib block JAK 1 and 2. They all are currently being tested for the therapeutic efficacy in refractory DM. Baricitinib was used successfully to treat a few children with JDM [145, 146], while tofacitinib was used to treat successfully 4 adult cases of DM [147], and ILD in some of the adults with MDA-5 [148]. Ruxolitinib, which affects circulating cytokine levels and regulates the activation of dendritic cells and T lymphocytes, improved the status of an adult with DM [149], and other dermatologic conditions responded as well [150]. The current recommendations are that women who are pregnant or breastfeeding should avoid this class of inhibitors [151].

### Lenabasum

An oral synthetic cannabinoid receptor type 2 agonist, lenabasum decreased pulmonary flares in patients with cystic fibrosis. This was accomplished by inhibiting the polarization of macrophages into M1 and reducing their secretion of IL-8 and TNF-α [152]. Lenabasum has been used successfully to treat skin diseases in patients with diffuse cutaneous systemic sclerosis [153]. Pruritus is a common feature of both adult and juvenile dermatomyositis, and mast cells have been identified in both symptomatic and apparently normal sun-protected skin [154]. The “itch” has been linked to increased skin IL-31 [155]. Several recent phase 2 trials have indicated increased function and decreased skin symptoms with use of this emerging drug.

### Other therapies

The treatment of cutaneous disease in JDM also includes avoidance of UVB exposure. The topical agents should contain UVA/B sunblock, such as titanium dioxide/oxide. A JDM child should avoid the sun during peak hours of 10–4 and wear photoprotective clothing—both purchased and home-rendered protective with the use of RIT SunGuard™. Windows can be covered with a UV blocking film, and photosensitizing drugs, such as antibiotics (sulfa, tetracyclines), non-steroidal anti-inflammatory drugs, and diuretics should be avoided. The itch can be treated topically and orally with antihistamines, as well as by employing a systemic therapy for the inflammatory process of JDM.

## Conclusion

The coordinated treatment of JDM is in its early stages, and there is great hope for more effective medications than those currently in use. The development of accessible serum biomarkers that could forecast disease flares would be very helpful and may aid in averting the premature cardiovascular compromise that is prevalent in adults who had JDM in childhood [156, 157].

## Figures and Tables

**Table 1.** Myositis Specific Antibodies (MSA) and other related myositis antibodies ^[48-51]^

Myositis Specific Antibodies						
Antibody Name	Frequency	Antigen Function	Muscle Disease	Skin Disease	Lung Disease	Histology muscle
Anti-TIF 1-γ	Adult: 7% Child: 18-30%	Transcription regulation	Amyopathic/mild severe	Photosensitive	None known	Varied, C5b-9 deposition macrophage ↑
Anit-NXP2	Adult: 2%-17% Child: 15-20%	Transcription regulation P53 activation, malignancy	Severe	Classic JDM rash	None known	Varied, C5b-9 deposition macrophage ↑
Anti-Mi2	Adult: 5%-10% Child: 4-10%	Transcription regulation	Generally mild	Classic JDM rash	None known	High severity score sarcolemma-complement deposition
Anti-MDA5	Adult: 1-30% Child: 7%	RNA-specifi helicase for host viral defense	Mild, may be absent (CADM)	Classic JDM rash	ILD dominant rapidly Progressive	Minimal change no C5b-9 deposition
Other Myositis Related Antibodies						
Antibody Name	Frequency	Antigen Function	Muscle Disease	Skin Disease	Lung Disease	Histology muscle
Anti-SRP	Adult: 2% Child: 2%	Targeting of protiens to endoplasmic reticulum	Severe at onset; very high CPK	Rash can occur atypical	ILD may occur	Moderately severe no C5b-9 deposition
Anti-ARS	Adult: 20-30% Child: 2%	Incorporates amino acids into their cognate tRNAs	Common-varies with tRNA	DM skin-rash Rapid progression	ILD domination	Perifasicular necrosis cytochrome oxidase ↓
Anti-HMGCR	Adult: 6% Child: 1%	Cholesterol biosynthesis	Severe; very high CPK	Rash can occur atypical	None known	Necrotizing myositis
Anti-SAE	Adult: 3% Child: 1%	Post-translation protein modification	Can develop later typically absent	Classic MD rash	None known	Necrotizing myositis^[50]^
Anti-Cn1A	Adult: 4-21% Child: 11-35%	Dephosphorylates Nucleoside Monophosphates	Adults ^[49]^: IBM pattern of weakness: Finger flexor quadriceps weakness	None known	None known	Inclusion body myositis

**Table 2.** Myositis Associated Antibodies (MAA) ^[48]^

Antibody Name	Frequency	Antigen Function	Muscle Disease	Skin Disease	Lung Disease	Other
Anti-Pm/Scl	Adults: 8% Child: 5%	RNA degradation	Frequent	DM rash Sclerodermatous rash	Lung dominant disease	Overlap disease SSc
Anti-U1RNP	Adult: 10% Child: 5%	splicing of mRNA	Frequent	uncommon	No known association	Overlap disease Mixed Connective Tissue Disease
Anti-Ro52	Adult: 25% Child: 6%	proteasome related degradation of target Protein	None known	Photosensitivity and rashes common	Associated	Associated with overlap disease
Anti-Ku	Adult:<1%	DNA repair	None known	None known	None known	Overlap Disease
